# Role of Neoadjuvant Chemotherapy on Pathological, Functional, and Survival Outcomes of Upper Tract Urothelial Carcinoma Patients: A Systematic Review and Meta-Analysis

**DOI:** 10.5152/tud.2024.23214

**Published:** 2024-01-01

**Authors:** Abdalla Ali Deb, Pragnitha Chitteti, Naufal Naushad, Wael Asaad, Steve Leung, Alice Hartley, Hosam Serag

**Affiliations:** 1 Department of Urology, James Cook University Hospital, Middlesbrough, UK; 2 Department of Urology, North Tees University Hospital, Stockton, UK; 3 Department of Urology, Western General Hospital, Edinburgh, UK; 4 Department of Urology, South Tyneside and Sunderland NHS Foundation Trust, Sunderland, UK; 5 Department of Urology, University Hospitals Birmingham, Birmingham, UK

**Keywords:** Glomerular filtration rate, neoadjuvant chemotherapy, pathological staging, survival, upper tract urothelial carcinoma

## Abstract

The role of neoadjuvant chemotherapy (NAC) in upper tract urothelial cancer (UTUC) is not yet confirmed. Therefore, we conducted this review to pool the available evidence in this regard. We analyzed 14 117 UTUC patients reported in 21 studies after searching 5 databases. The NAC was administered in 1983 patients and the remaining 12 134 controls underwent radical nephroureterectomy (RNU) alone. Efficacy endpoints included pathological, functional, and survival outcomes. Safety was determined by overall and grade 3-4 complications. For dichotomous outcomes, the log odds ratio (logOR) was pooled, and for continuous variables, the crude mean difference was calculated along with its 95% CI. The NAC was associated with 10% complete pathological response (CPR), 42% pathological downstaging, 31% post-NAC advanced disease (pT3-4), 6% positive surgical margin, 18% lymph node metastasis (pN+), 24% lymphovascular invasion, and 29% mortality and recurrence at 5 years. Compared to controls, NAC resulted in increased risk of CPR [logOR = 1.67; 95% CI, 0.11-3.23] and downstaging [logOR = 1.30; 95% CI, 0.41-2.18] and reduced risk of advanced disease [logOR = −0.81; 95% CI, −1.51-−0.11]. Renal function did not improve from baseline; however, it increased significantly after RNU. The NAC was associated with good survival/low mortality in the short term, with a sustained increase over time. Overall and grade 3-4 complications occurred in 25% and 7% of patients, respectively. Our findings support the potential benefits of NAC in enhancing pathological outcomes and possibly improving survival in UTUC patients undergoing RNU. The variability in response and associated complications underscore the importance of careful patient selection and tailored treatment approaches.

**Main Points**

- Neoadjuvant chemotherapy results in pathological downstaging in approximately half the treated population.
- Neoadjuvant chemotherapy is associated with high mortality and recurrence in the long-term.
- Neoadjuvant chemotherapy does not affect renal function; however, one-fourth of the population experiences complications.

## Introduction

Upper tract urothelial carcinoma (UTUC), encompassing the tumors of the renal pelvis and ureter, represents a relatively rare subset of urothelial cancers, accounting for approximately 5%-10% of all urothelial malignancies.^1^ Despite its infrequency when compared to bladder cancer, UTUC is often diagnosed at a more advanced stage and is associated with a poorer prognosis when compared to transitional cell carcinoma (TCC) of the lower urinary tract.^2^ The standard treatment for localized UTUC is radical nephroureterectomy (RNU); however, the high rate of recurrence and further disease progression necessitate the exploration of additional therapeutic strategies to improve patient outcomes.^3^

Neoadjuvant chemotherapy (NAC), the administration of systemic chemotherapy before definitive surgical intervention, has emerged as a potential strategy to enhance the management of UTUC.^4^ The rationale behind NAC includes the potential to downstage the tumor, eradicate micrometastatic disease, and improve overall survival. While NAC has become a standard of care in muscle-invasive bladder cancer,^5^ its role in UTUC remains controversial and is supported by a limited number of studies with varying methodologies and outcomes.

Previous systematic reviews and meta-analyses have attempted to clarify the impact of NAC on UTUC,^6-8^ but the results have been inconclusive, partially due to the heterogeneity of the included studies and the limited number of randomized controlled trials. Furthermore, the safety and tolerability of NAC in the UTUC population, which often presents with associated comorbidities and compromised renal function, is a critical factor to be considered, warranting a thorough investigation.

This systematic review and meta-analysis aim to synthesize the current evidence on the efficacy and safety of neoadjuvant chemotherapy in patients with UTUC. By rigorously evaluating and summarizing the findings from original research articles, this study seeks to provide a comprehensive and up-to-date analysis to facilitate informed clinical practice and guide future research in the management of UTUC.

## Material and Methods

### Design and Population

This research was conducted in line with the guidelines provided for systematic reviews and meta-analyses in the Preferred Reporting Items for Systematic Reviews and Meta-Analyses (PRISMA) checklist. The design of this review followed the PICOS (Population, Intervention, Comparison, Outcome, and Study) framework.^9^ We included patients with UTUC who received NAC prior to RNU. The presence of a comparison group was not mandated; however, we included comparative studies that included patients who underwent only RNU without prior NAC as a control group. Pathological, functional, and survival outcomes were measured. Randomized and non-randomized (observational) studies of intervention were considered.

### Literature Search

On September 11, 2023, a literature search was performed across 4 databases (PubMed, Scopus, Web of Science, and Cochrane Registry of Randomized Controlled Trials) and one registry (Google Scholar). In the latter, only the first 200 citations were searched as per the recommendations.^10^ A list of relevant keywords and medical terms was used, which were adjusted accordingly as per the searched database. These terms included were “UTUC” or “upper tract urothelial carcinoma” and “nephroureterectomy” and “neoadjuvant chemotherapy” or “neo-adjuvant chemotherapy.” The full search query is provided in Supplementary Table 1.

### Selection Criteria

Studies meeting the following criteria were included: (a) experimental or observational studies; (b) investigating UTUC patients who received NAC (regardless of the presence or absence of a comparison group); and (c) reporting pathological, functional, or survival outcomes or complications. Studies were excluded if: (a) the sample size was <20 patients; (b) non-original research (i.e., reviews); (c) duplication; or (d) irrelevant outcomes. We did not exclude studies based on the original language of publication.

### Outcomes Measures

Outcomes were divided into 4 main categories: pathological, functional, and survival outcomes; and safety profile. Pathological outcomes included complete pathological response (CPR), partial pathological response (PPR), pathological downstaging, post-NAC advanced disease (pT3-4), positive surgical margin (PSM), lymph node metastasis (pN+), and lymphovascular invasion (LVI). Survival outcomes included mortality/death, disease recurrence, overall survival (OS), cancer-specific survival (CSS), progression-free survival (PFS), and recurrence-free survival (RFS), all of which were subdivided based on the follow-up time. Functional outcomes included the estimated glomerular filtration rate (eGFR), which was compared between the pre-NAC, post-NAC, post-RNU, and control groups. The safety profile was reported in terms of overall complications and grade 3-4 complications. Of note, in each of these outcomes, the prevalence across the NAC group was calculated, and a comparison was made with the control group.

### Study Selection

Studies identified from the literature search were imported into EndNote software, where duplicates were identified and excluded. After that, the remaining studies were exported into an Excel sheet for screening. The screening process was done by 2 investigators simultaneously over 3 different phases: title, abstract, and full-text selection. Any differences between them were resolved by consulting the senior author.

### Data Extraction and Methodological Quality Assessment

The senior author used Microsoft Excel to design the data extraction sheet, which consisted of 3 worksheet tabs. The first part covered studies’ and patients’ characteristics, including the study design, year of publication, sample size, included groups (NAC and control), age, gender, and follow-up period. The second part covered the outcomes of interest. The third part covered the methodological quality [risk of bias (ROB)] of included studies. Since no randomized trials were identified, the Newcastle–Ottawa Scale (NOS) for observational studies was used for this purpose. This tool assesses the methodology of each study based on selection (4 points), comparability (2 points), and outcome (3 points). The data extraction and quality assessment were both performed by 2 investigators, and the senior author was consulted whenever needed to resolve any differences between them in this regard.

### Data Analysis

Data analysis was performed using STATA Software,Stata Corp LLC, USA, version 18.0. To calculate the prevalence of our outcomes in the NAC group, the metaprop command was used, where the effect size (ES) of all studies was pooled. The fixed-effects and random-effects models were selected based on the presence of statistical heterogeneity. If heterogeneity was significant (*I* ^2^ > 50% with a *P*-value <.05), the random-effects model was chosen.

For functional outcomes (continuous in nature), the crude mean difference (MD) was calculated using the inverse variance method when heterogeneity was absent and using the restricted maximum likelihood (REML) method when heterogeneity was present. For other outcomes (dichotomous in nature), the log odds ratio (logOR) was calculated using the Mantel–Haenszel method and the REML method when heterogeneity was absent or present, respectively.

Whenever statistical heterogeneity was encountered, a leave-one-out sensitivity analysis was done to determine if the reported effect estimate was driven by a particular study. A *P *-value of <.05 was deemed statistically significant.

## Results

### Database Search Results

**Figure 1.**
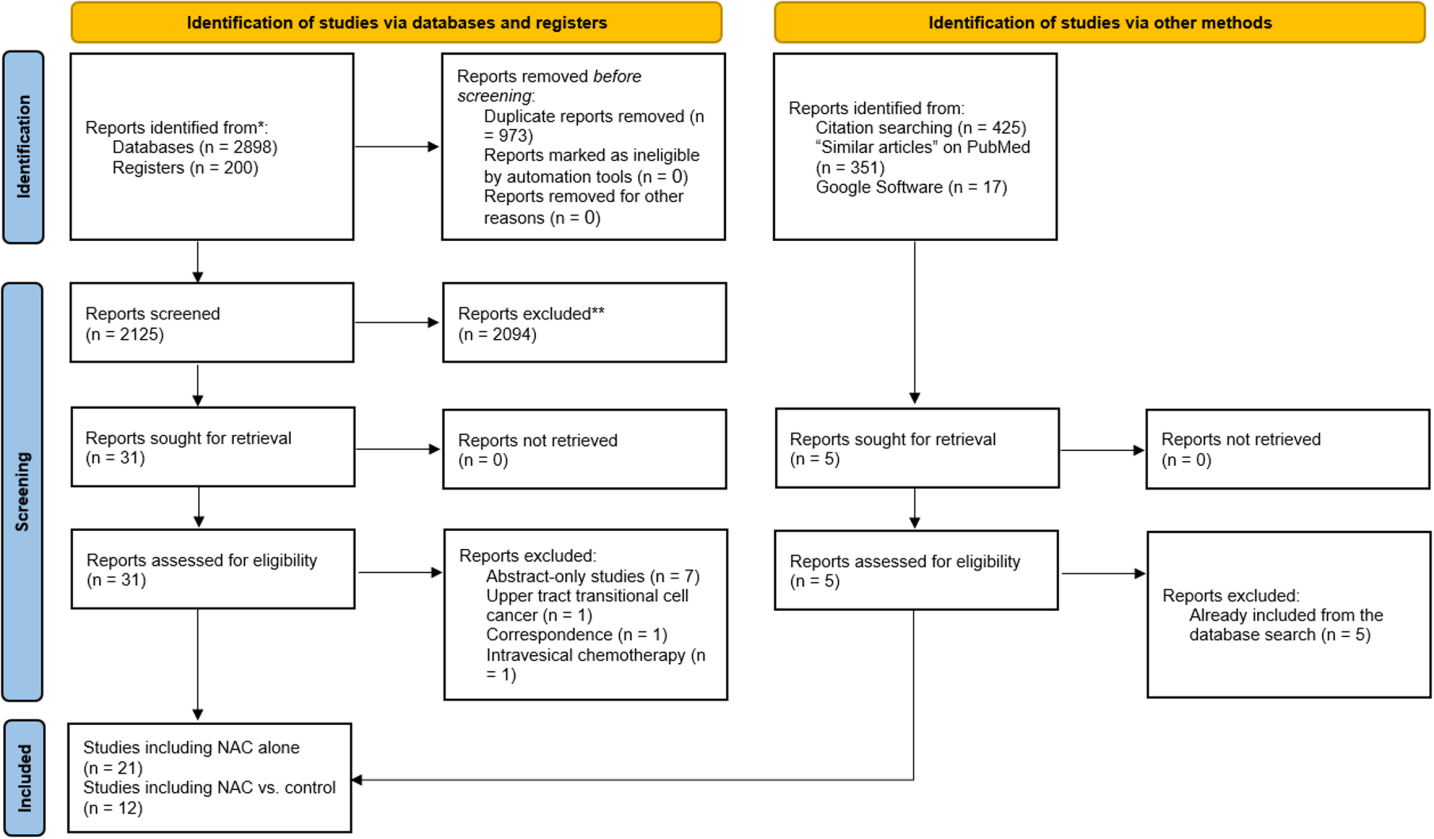
PRISMA diagram showing the results of the literature search and study selection processes.

The detailed process of literature search and study selection is described in Figure 1. The literature search yielded 3098 results, of which 973 were duplicates. The titles of 2125 reports were screened, of which 31 passed to the full-text screening phase. Ten studies were excluded because they were either only conference abstracts (7 studies), discussed intravesical application of chemotherapy (1 study), non-original in design (correspondence, 1 study), or included patients with transitional cell cancer (1 study). Finally, 21 studies were included in the data synthesis phase (21 reporting NAC, of which 12 included a control “surgery-only” group).^11-31^ No additional studies were identified after the manual search. Importantly, one article was published in French and was translated with the help of a bilingual (French–English) colleague.

### Characteristics of Included Studies

The characteristics of the included studies and the patients are presented in Table 1. Nineteen studies were retrospective cohort in design, and only 2 studies were phase II clinical trials. No randomized trials have been published so far. Most studies were conducted in the USA (11 studies), followed by Japan (5 studies). A total of 1983 UTUC patients who received NAC and 12 134 patients in the control group were analyzed. Patients’ age and gender, the use of adjuvant chemotherapy after both NAC and RNU, and the tumor location are summarized in Table 1. The regimen, the number of doses, and the number of cycles of administered NAC are summarized in Table 2.

### Methodological Quality of Included Studies

The summary of the scoring of each study based on the NOS is presented in Table 3. The methodological quality of 12 of the included studies was deemed good, while the remaining 10 studies were graded as having fair quality. The domain that was most affected was comparability, given the lack of a comparison group in 10 of included studies.

### Pathological Outcomes of Neoadjuvant Chemotherapy

#### Complete pathological response:

**Figure 2.**
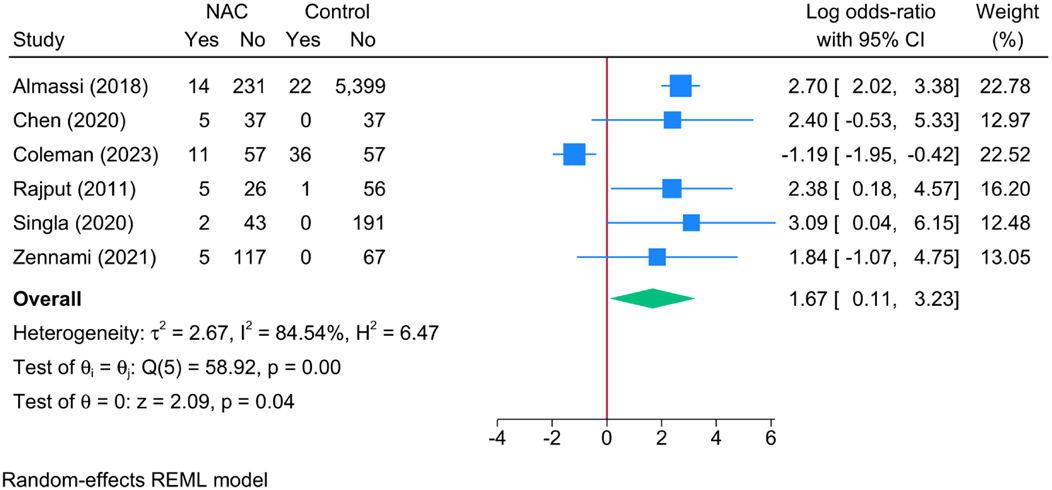
Forest plot showing the comparative risk of complete pathological response between the neoadjuvant chemotherapy and control groups

**Supplementary Figure 1.**
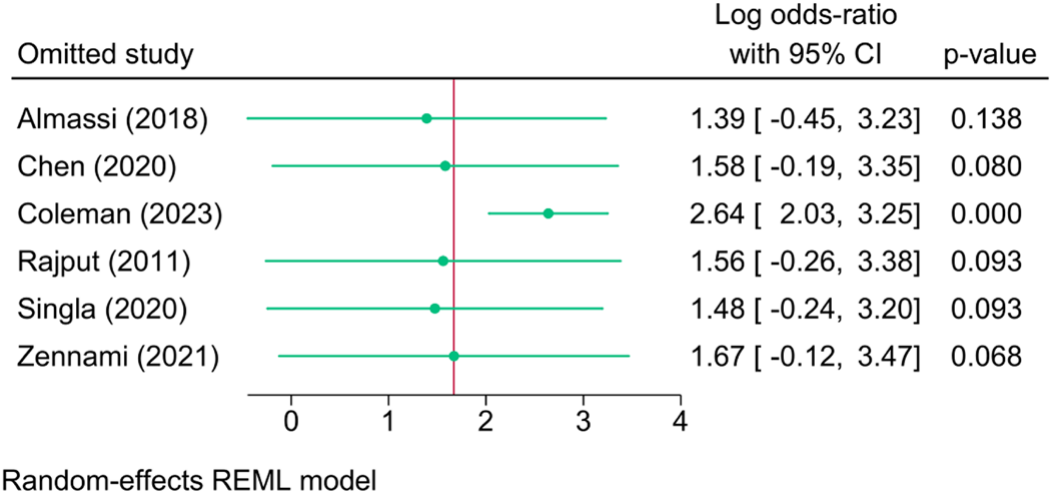
Leave-one-out sensitivity analysis of complete pathological response.

Sixteen studies, comprising 1595 patients, reported the rate of CPR among patients who received NAC. The meta-analysis revealed an overall rate of 10% [95% CI, 8-13]. Compared to the control group, the NAC group was associated with significantly higher odds of CPR [6 studies, logOR = 1.67; 95% CI, 0.11-3.23] (Figure 2). Significant heterogeneity was observed [*I* ^2^ = 84.54%, *P *= .001]; however, the leave-one-out sensitivity analysis revealed that the study by Coleman et al^14^ was the main contributor to heterogeneity (Supplementary Figure 1).

#### Partial pathological response:

Four studies [452 patients] reported the rate of PPR in NAC patients. The meta-analysis revealed an overall rate of 48% [95% CI, 44-53]. However, there was no comparison made with regards to the control group.

#### Downstaging:

**Figure 3.**
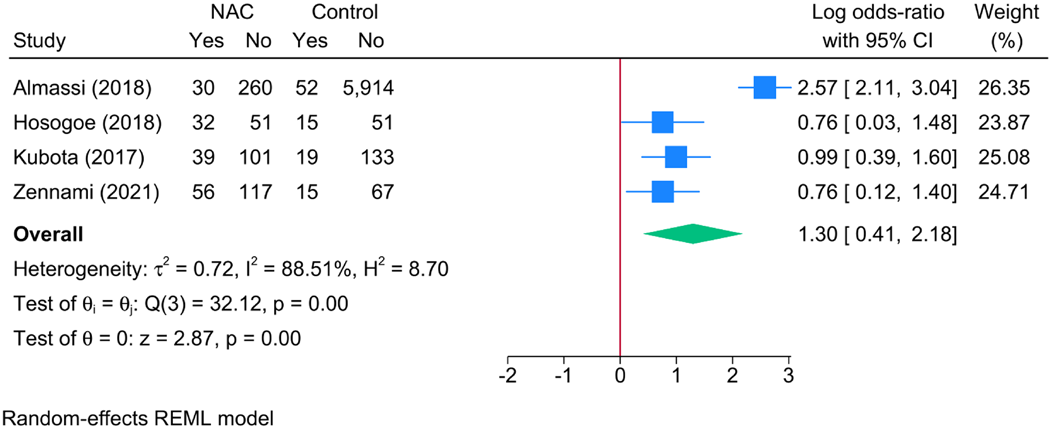
Forest plot showing the comparative risk of pathological downstaging between the neoadjuvant chemotherapy and control groups.

**Supplementary Figure 2.**
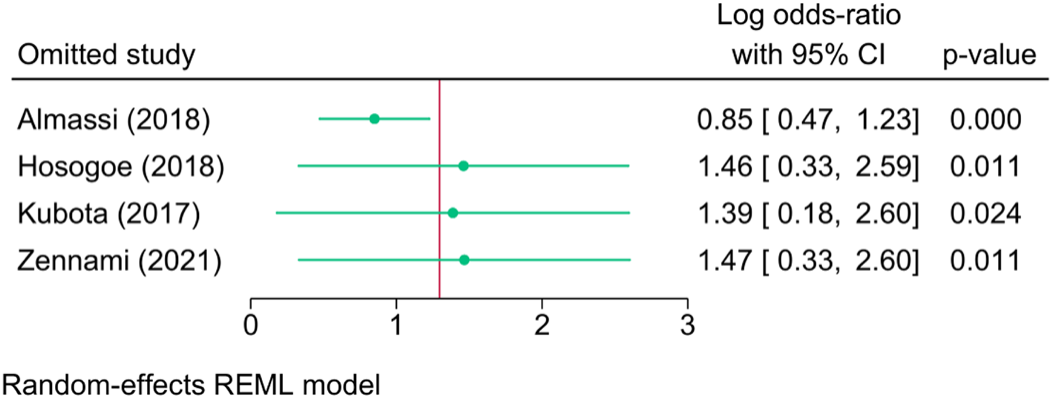
Leave-one-out sensitivity analysis of pathological downstaging.

Eight studies [1102 patients] reported the rate of pathological downstaging in NAC patients. The meta-analysis revealed an overall rate of 42% [95% CI, 29-54]. Compared to the control group, patients who underwent NAC have demonstrated significantly higher odds of pathological downstaging [4 studies, logOR = 1.30; 95% CI, 0.41-2.18] (Figure 3). Significant heterogeneity was observed [*I* ^2^ = 88.51%, *P *= .001], however, the leave-one-out sensitivity analysis did not show any significant change in the reported effect (Supplementary Figure 2).

#### Post-NAC advanced disease (pT3 or pT4):

**Figure 4.**
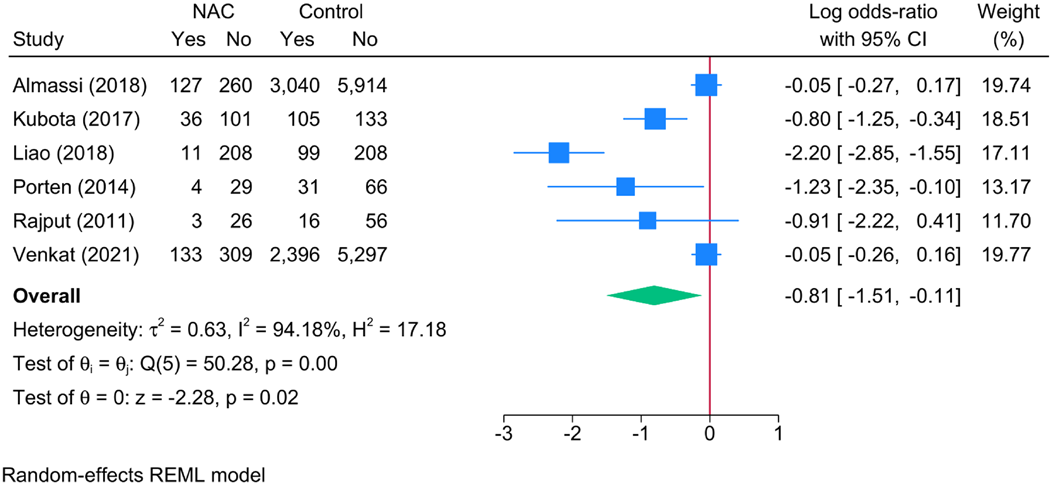
Forest plot showing the comparative risk of advanced disease between the neoadjuvant chemotherapy and control groups.

**Supplementary Figure 3.**
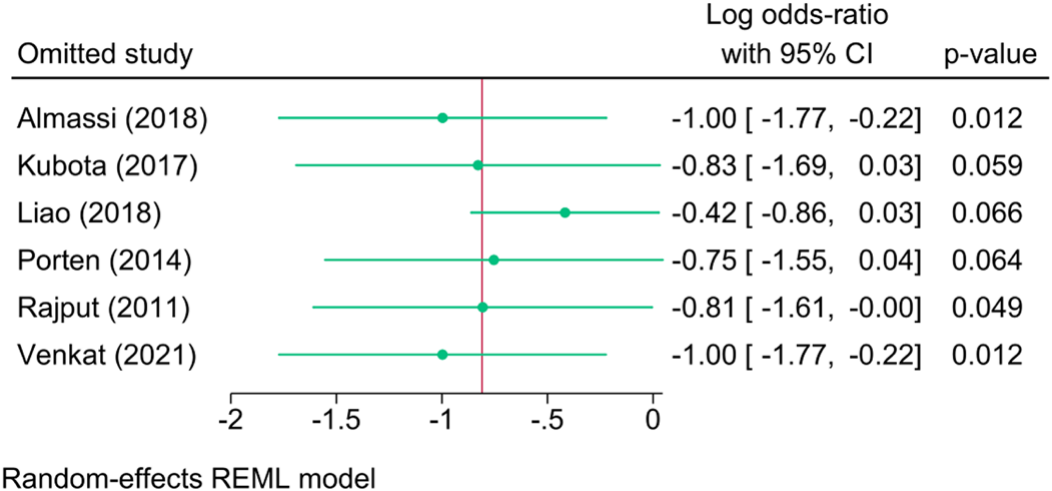
.Leave-one-out sensitivity analysis of post-neoadjuvant chemotherapy advanced disease.

Nine studies [1155 patients] reported the rate of pT3-4 in NAC patients. The meta-analysis revealed an overall rate of 31% [95% CI, 20-42]. Compared to the control group, the NAC group was associated with a significantly lower risk of advanced disease [6 studies, logOR = −0.81; 95% CI, −1.51-0.11] (Figure 4). Significant heterogeneity was observed [*I* ^2^ = 94.18%, *P *= .001]; however, the leave-one-out sensitivity analysis revealed that the studies of Almassi et al^12^ and Venkat et al^29^ were the main contributors to the observed heterogeneity (Supplementary Figure 3).

#### Positive surgical margin:

**Supplementary Figure 4.**
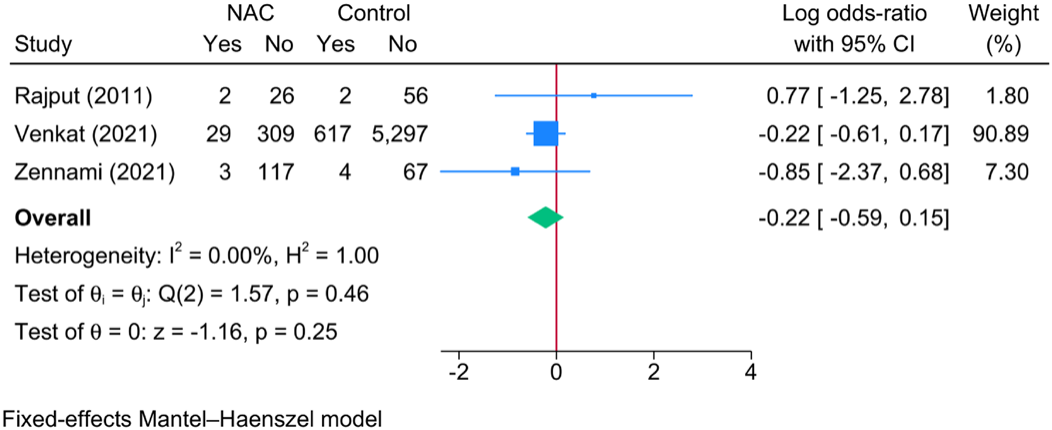
Forest plot showing the comparative risk of positive surgical margin between the neoadjuvant chemotherapy and control groups.

Seven studies [917 patients] reported the rate of PSM in NAC patients. The meta-analysis revealed an overall rate of 6% [95% CI, 3-8]. Compared to the control group, NAC was not associated with a significant difference in the risk of PSM [3 studies, logOR = −0.22; 95% CI, −0.59-0.15] (Supplementary Figure 4). No heterogeneity was observed [*I* ^2^=0%, *P *= .46].

#### Lymph node metastasis:

**Supplementary Figure 5.**
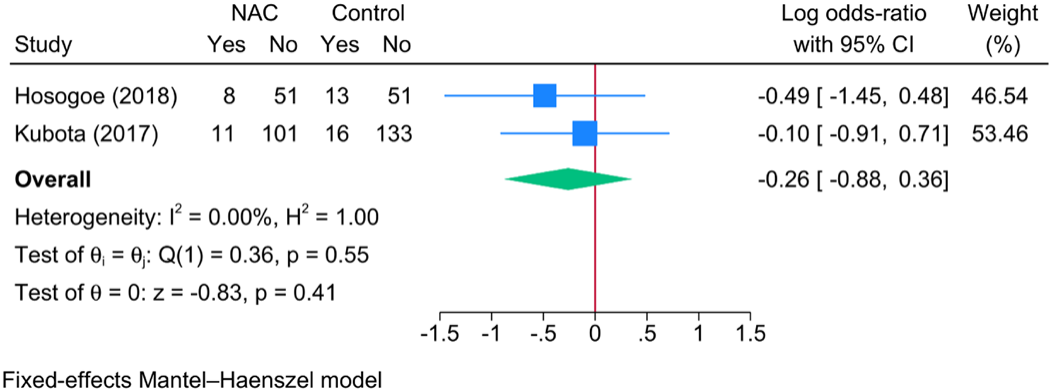
.Forest plot showing the comparative risk of lymph node metastasis (pN+) between the neoadjuvant chemotherapy and control groups.

Six studies [923 patients] reported the rate of pN+ in NAC patients. The meta-analysis revealed an overall rate of 18% [95% CI, 13-24]. Compared to the control group, NAC was not associated with a significant difference in the risk of pN+ [2 studies, logOR = −0.26; 95% CI, −0.88: 0.36] (Supplementary Figure 5). No heterogeneity was observed [*I* ^2^ = 0%, *P *= .46].

#### Lymphovascular invasion:

**Figure 5.**
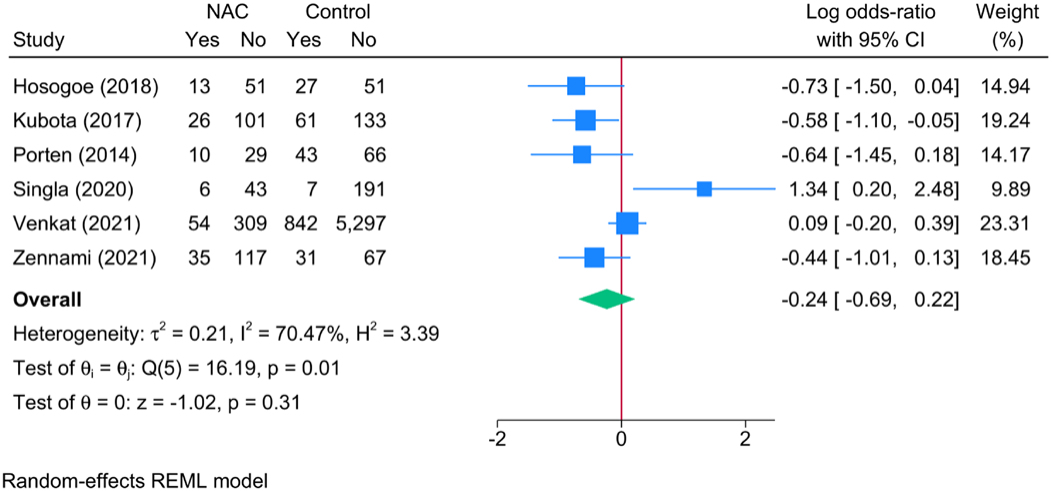
Forest plot showing the comparative risk of lymphovascular invasion between the neoadjuvant chemotherapy and control groups.

Seven studies [756 patients] reported the rate of LVI in NAC patients. The meta-analysis revealed an overall rate of 24% [95% CI, 19-29]. Compared to the control group, NAC was not associated with a significant difference in the risk of LVI [6 studies, logOR = −0.24; 95% CI, −0.69-0.22] (Figure 5). Significant heterogeneity was observed [*I* ^2^ = 70.47%, *P *= .01], however, the leave-one-out sensitivity analysis did not reveal any significant change in the reported estimate.

### Survival Outcomes of NAC

#### Mortality:

**Figure 6.**
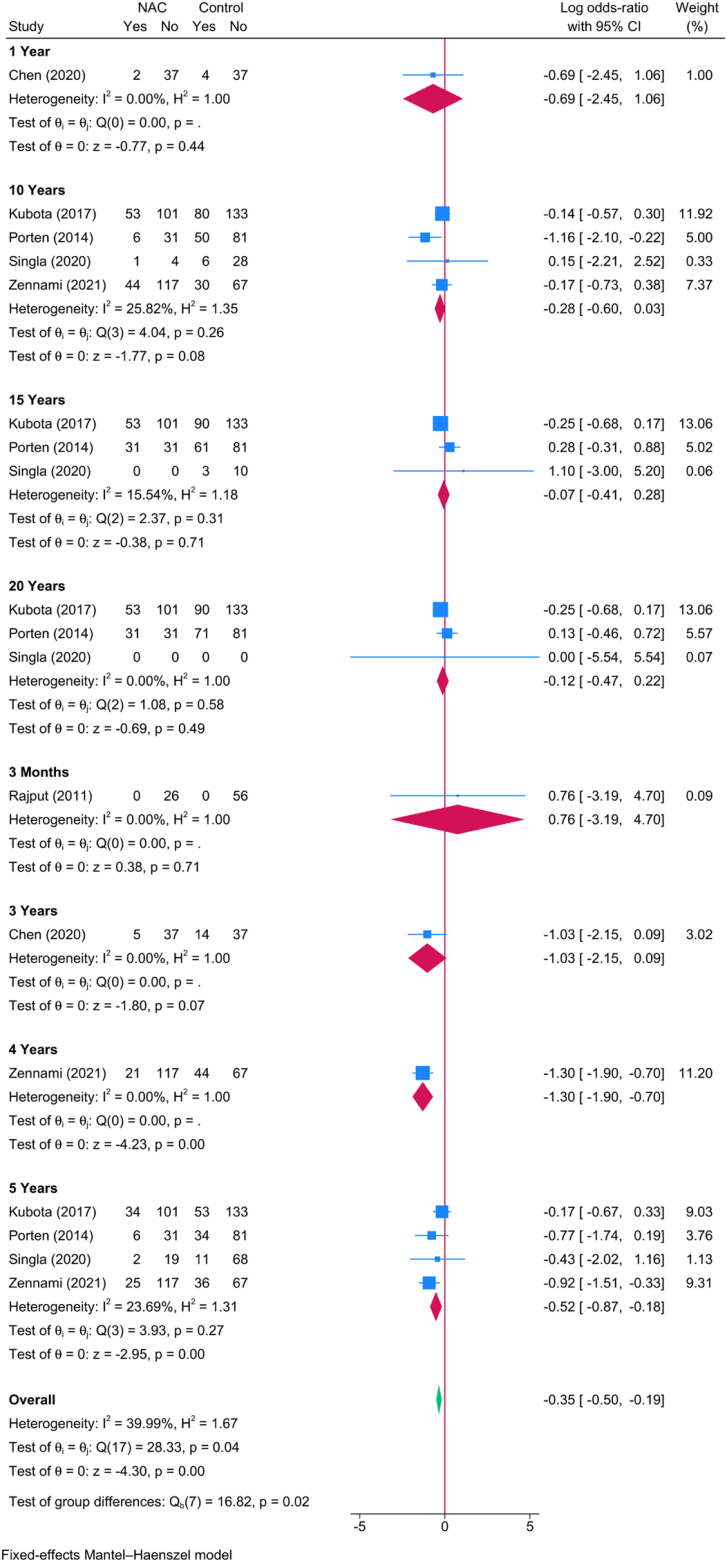
Forest plot showing the comparative risk of mortality between the neoadjuvant chemotherapy and control groups, stratified by follow-up period.

The rate of post-NAC mortality was lowest at 5 years [ES = 29%; 95% CI, 17-42%]. This rate increased gradually, demonstrating the highest rate at 20 years [ES = 76%; 95% CI, 31-100]. Compared to the control group, NAC was associated with a significant reduction in the risk of mortality at 5 years [4 studies, logOR = −0.52; 95% CI, −0.87-−0.18]. However, this difference became insignificant over the long term (Figure 6).

#### Disease recurrence:

**Figure 7.**
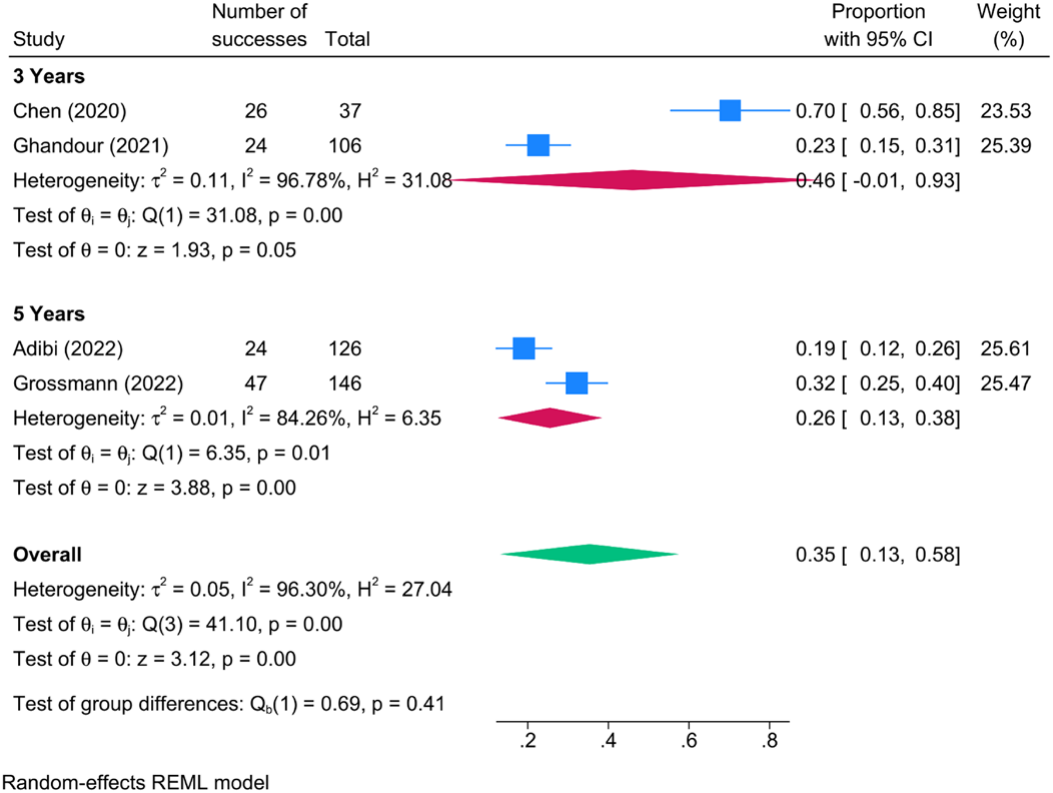
Forest plot showing the prevalence of recurrence following neoadjuvant chemotherapy stratified by follow-up period.

The rate of post-NAC mortality was the lowest at 5 years [ES = 29%; 95% CI, 17-42]. This rate increased gradually, hitting the highest rate at 20 years [ES = 76%; 95% CI, 31-100]. Compared to the control group, the NAC group was associated with a significant reduction in the risk of mortality at 5 years [4 studies, logOR = −0.52; 95% CI, −0.87-−0.18] (Figure 7). However, this difference became insignificant over the long-term follow-up.

#### Overall survival:

**Figure 8.**
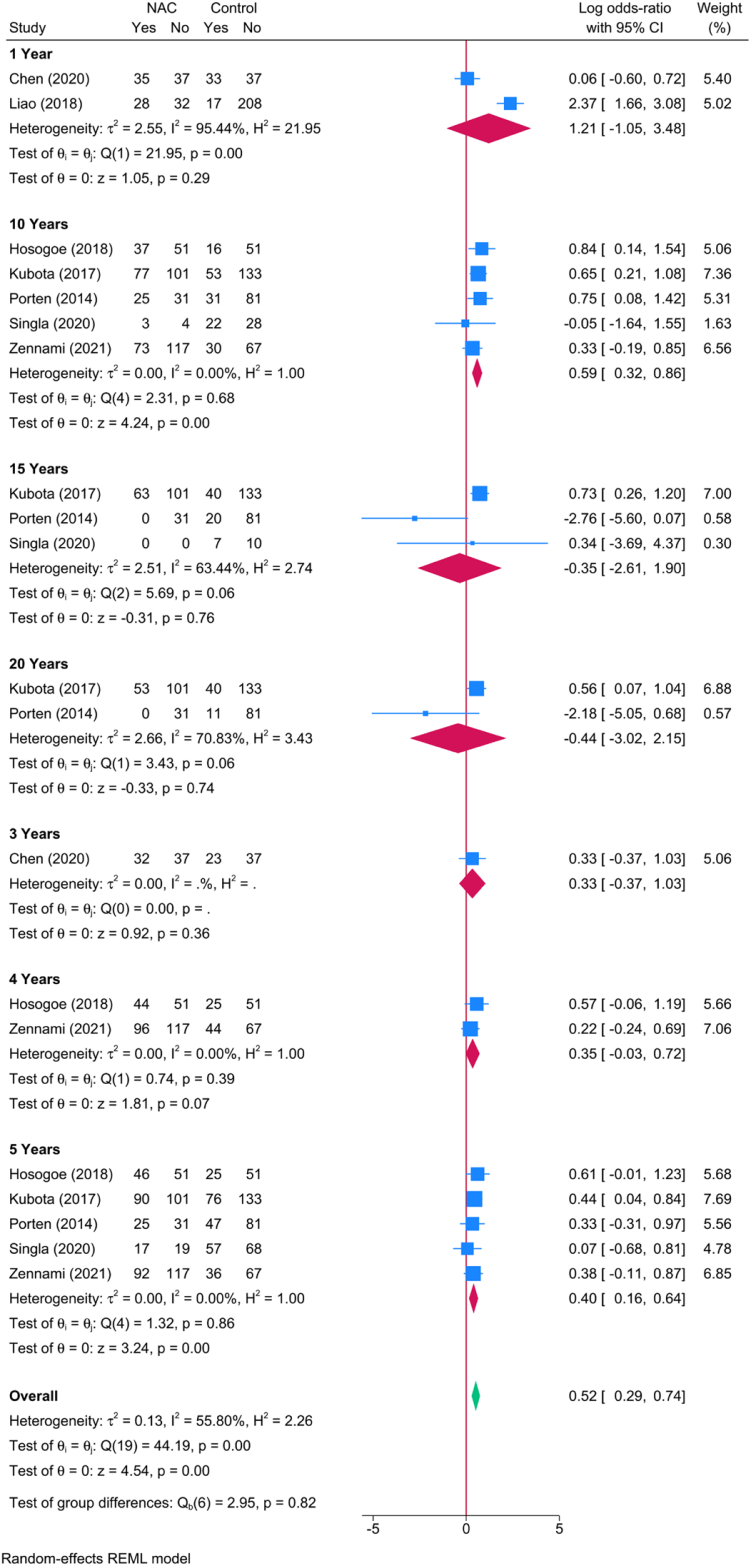
Forest plot showing the comparative overall-survival between the neoadjuvant chemotherapy and control groups, stratified by follow-up period.

The rate of post-NAC OS was the highest at 1 year [ES = 90%; 95% CI, 85-95%], and it declined steadily over time until it became the lowest at 20 years [ES = 27%; 95% CI, 0-77%] of follow-up. Compared to the control group, the NAC cohort was associated with significantly higher odds of OS in 5 years [5 studies, logOR = 0.40; 95% CI, 0.16-0.64] and 10 years of follow-up [5 studies, logOR = 0.59; 95% CI, 0.32-0.86] (Figure 8).

#### Cancer-specific survival:

**Figure 9.**
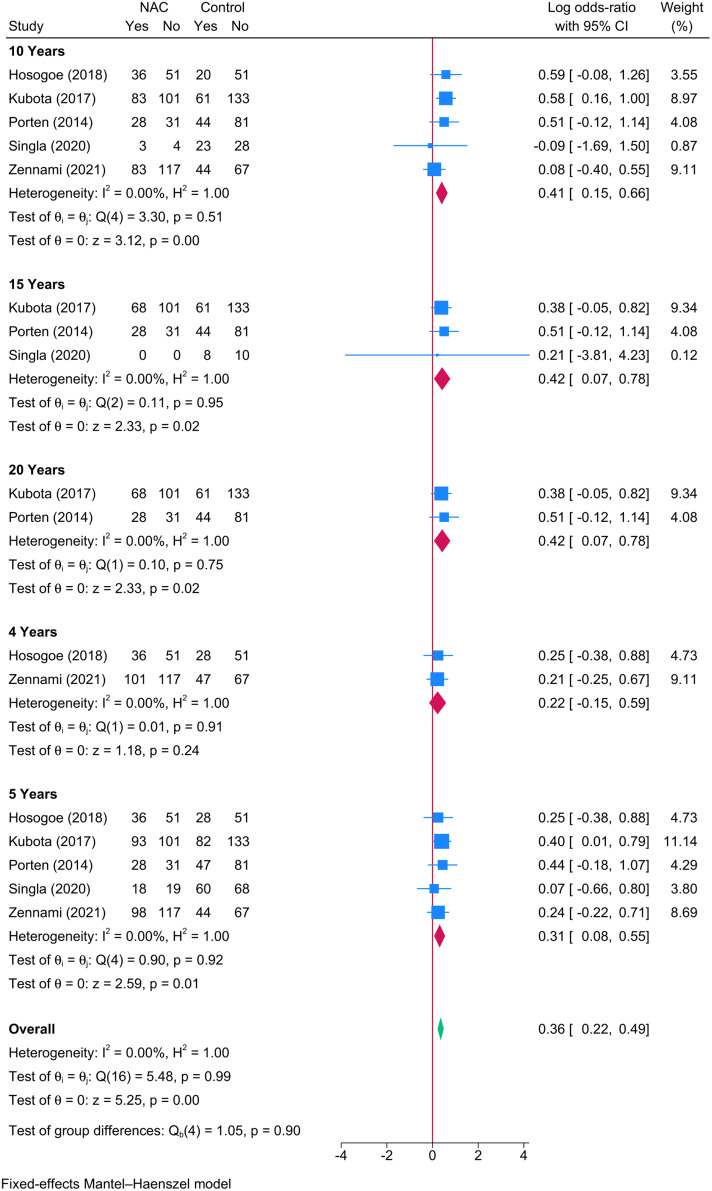
Forest plot showing the comparative cancer-specific survival between the neoadjuvant chemotherapy and control groups, stratified by follow-up period.

The rate of post-NAC CSS was similar across all follow-up periods: 5 years [7 studies, ES = 80%; 95% CI, 67-92], 10 years [5 studies, ES = 79%; 95% CI, 70-87], 15 years [2 studies, ES = 79%; 95% CI, 56-100], and 20 years [2 studies, ES = 79%; 95% CI, 56-100]. Compared to the control group, NAC was associated with significantly higher odds of CSS in 5 years [5 studies, logOR = 0.31; 95% CI, 0.08-0.55], 10 years [5 studies, logOR = 0.41; 95% CI, 0.15-0.66], 15 years [3 studies, logOR = 0.42; 95% CI, 0.07-0.78], and 20 years [2 studies, logOR = 0.42; 95% CI, 0.07-0.78] (Figure 9).

#### Progression-free survival:

**Figure 10.**
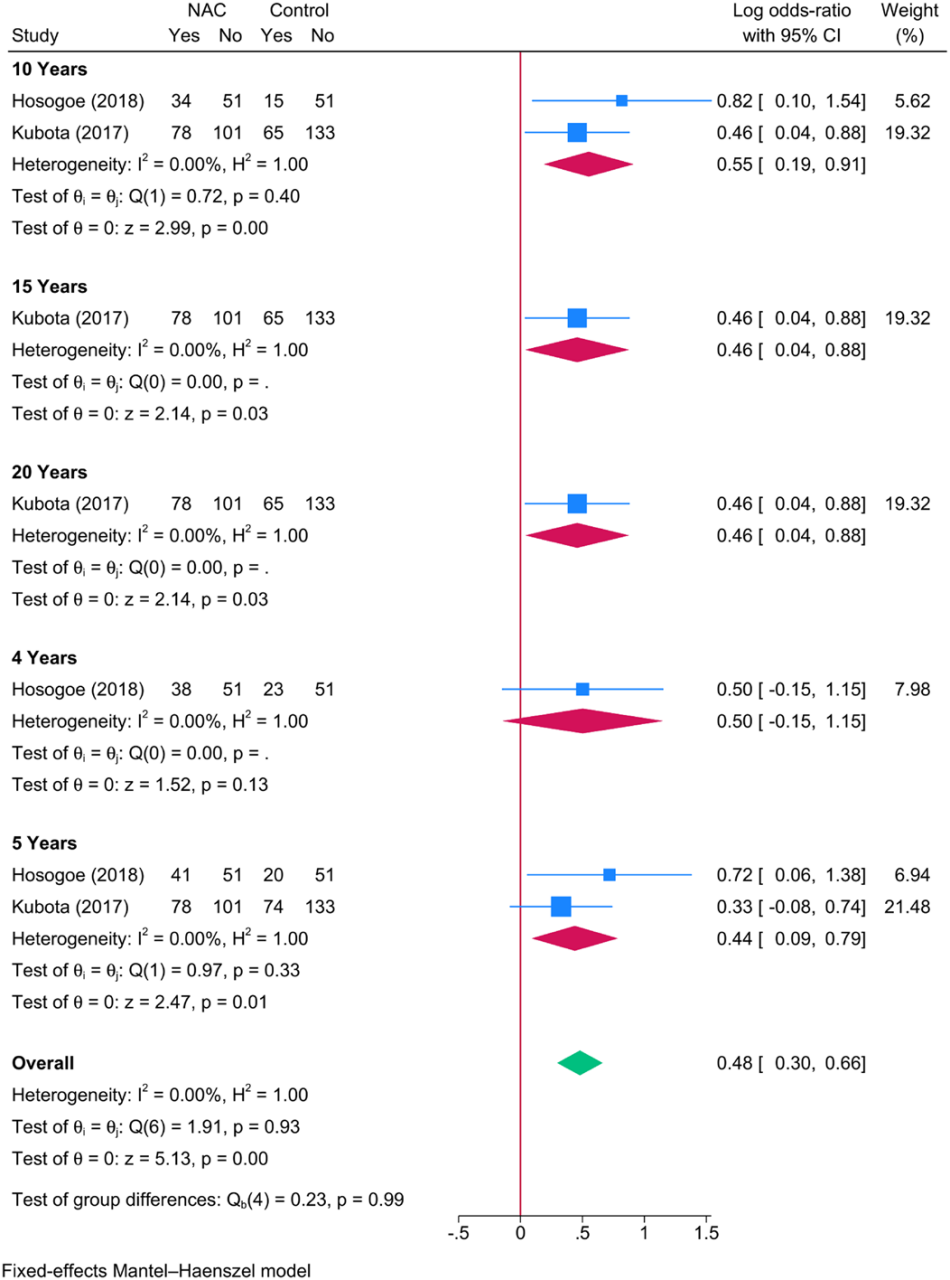
Forest plot showing the comparative progression-free survival between the neoadjuvant chemotherapy and control groups, stratified by follow-up period.

The rate of post-NAC PFS was lower in 5 years [ES = 69%; 95% CI, 53-85] and higher in 10 years [ES = 73%; 95% CI, 63-83] of follow-up. Compared to the control group, the NAC group was associated with significantly higher odds of PFS in 5 years [2 studies, logOR = 0.44; 95% CI, 0.09-0.79] and 10 years of follow-up [2 studies, logOR = 0.55; 95% CI, 0.19-0.91] (Figure 10).

#### Recurrence-free survival:

**Supplementary Figure 6.**
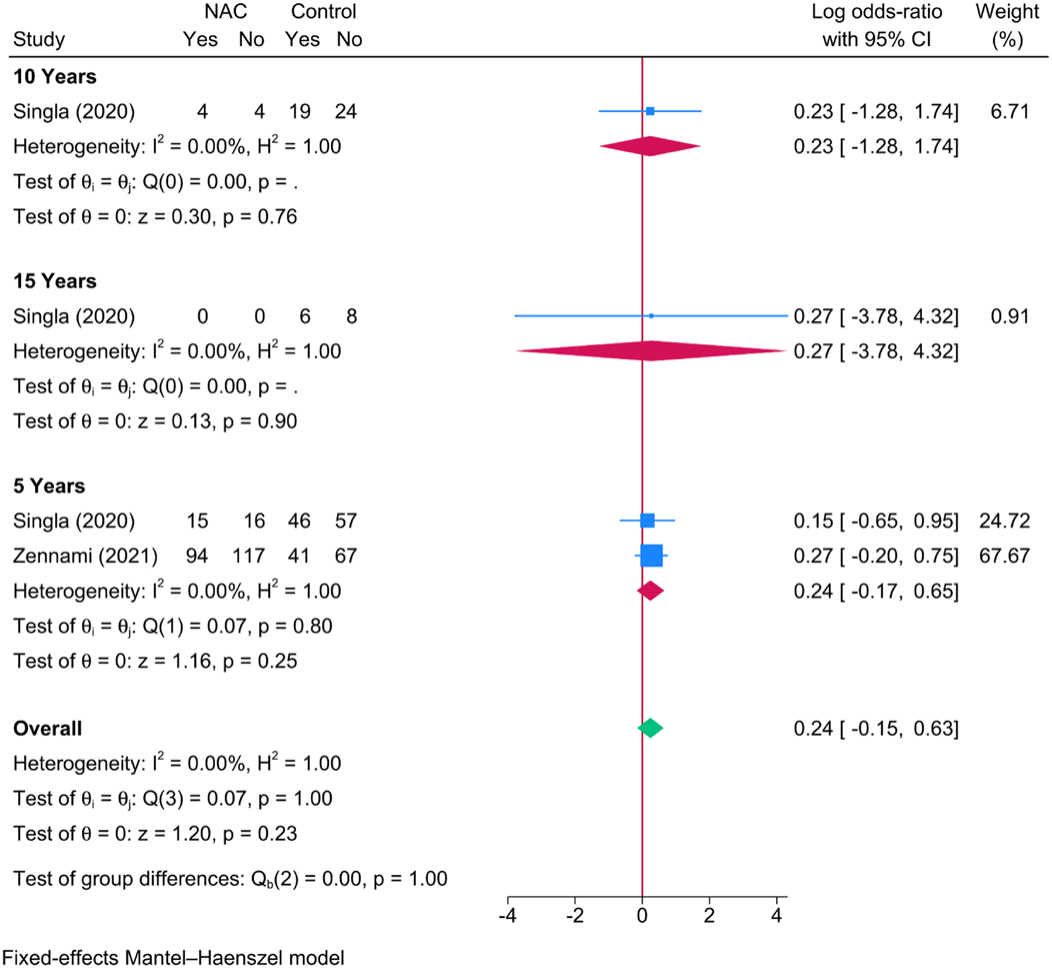
Forest plot showing recurrence-free survival between the neoadjuvant chemotherapy and control groups, stratified by follow-up period.

The rate of post-NAC RFS was lower in 5 years [4 studies, ES = 69%; 95% CI, 53-85%] and higher in 10 years [2 studies, ES = 73%; 95% CI, 63-83%] of follow-up. No significant difference was noted between the NAC and control groups with regards to the 5-year RFS [2 studies, logOR = 0.24; 95% CI, −0.17-0.65]. Other timepoints (10 and 15 years) were represented by only one study, and therefore, meaningful interpretations could not be made (Supplementary Figure 6).

### Functional Outcome—Renal Function (Estimated Glomerular Filtration Rate)

#### Pre-NAC vs. post-NAC:

**Supplementary Figure 7.**
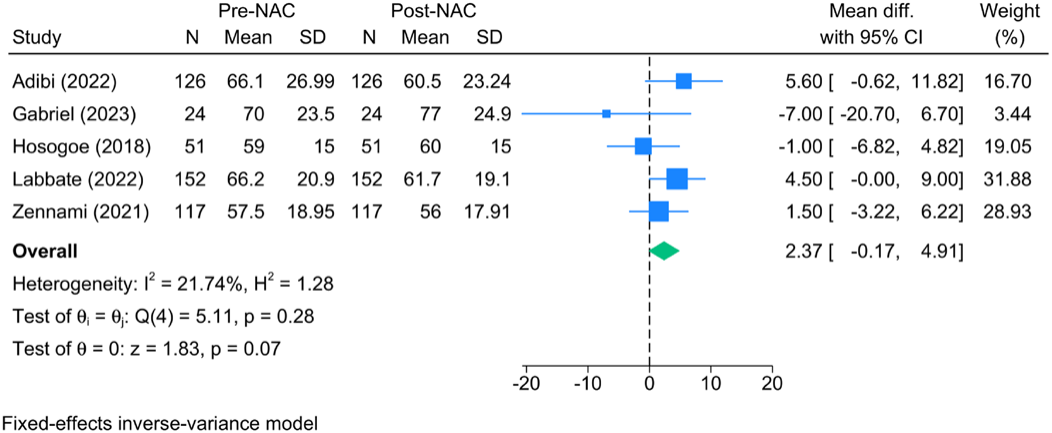
Forest plot showing the difference in eGFR levels post-neoadjuvant chemotherapy from the baseline.

Five studies compared the eGFR levels before and after administering NAC. The use of NAC did not result in any significant change in eGFR compared to pre-NAC levels [MD = 2.37; 95% CI, −0.17-4.91]. No significant heterogeneity was observed [*I* ^2^ = 21.74%, *P *= 0.28] (Supplementary Figure 7).

#### Pre-NAC vs. post-RNU:

**Supplementary Figure 8.**
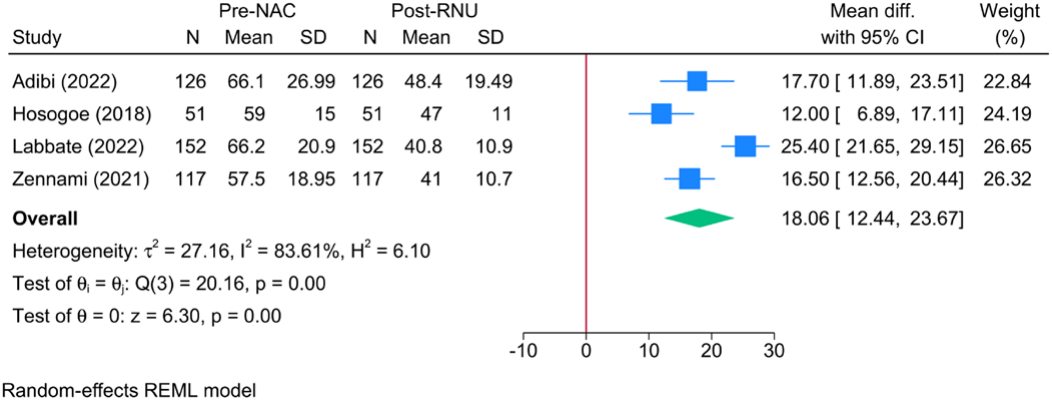
Forest plot showing the difference in eGFR levels post-radical nephroureterectomy from the baseline.

**Supplementary Figure 9.**
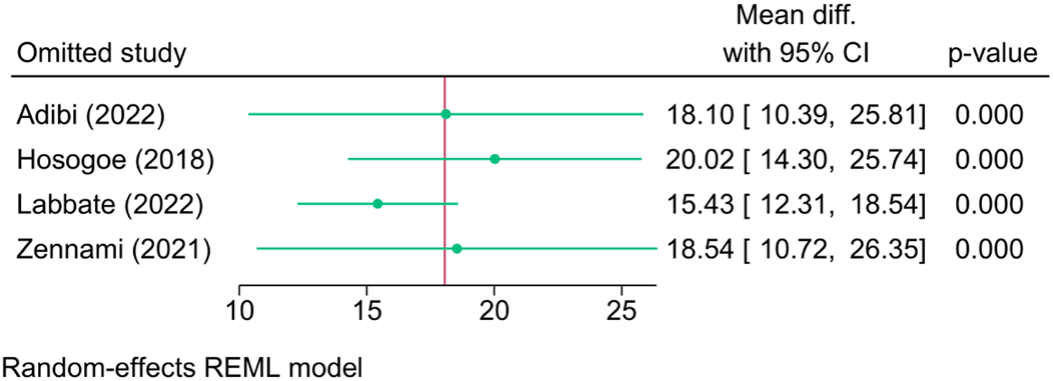
Leave-one-out sensitivity analysis between baseline and post-radical nephroureterectomy eGFR levels.

Four studies compared the eGFR levels between pre-NAC and post-RNU. Radical nephroureterectomy results in statistically significant increase in eGFR compared to pre-NAC levels [MD = 18.06; 95% CI, 12.44-23.67] (Supplementary Figure 8). We observed statistically significant heterogeneity [*I* ^2^ = 83.61%, *P *= .001]; however, the leave-one-out sensitivity analysis revealed no significant change in the reported effect estimate (Supplementary Figure 9).

#### Post-NAC vs. post-RNU:

**Supplementary Figure 10.**
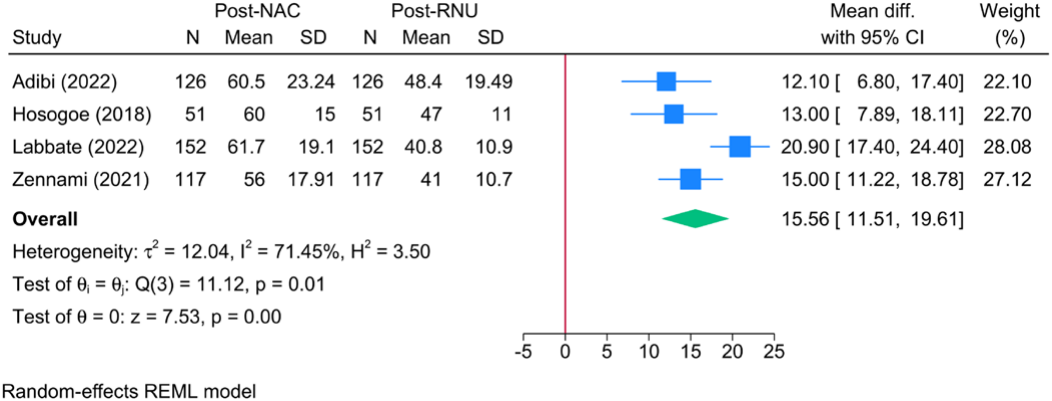
Forest plot showing the difference between post-neoadjuvant chemotherapy and post-radical nephroureterectomy eGFR levels.

**Supplementary Figure 11.**
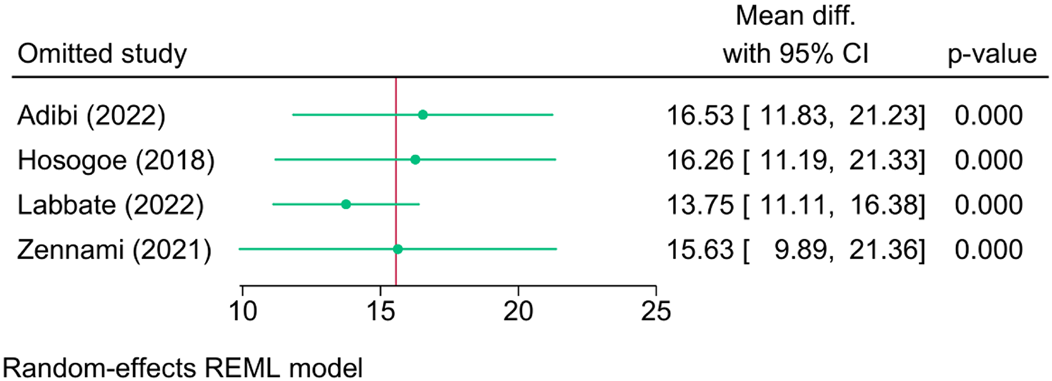
Leave-one-out sensitivity analysis of post-neoadjuvant chemotherapy and post-radical nephroureterectomy eGFR levels.

Four studies compared the eGFR levels between post-NAC and post-RNU. Radical nephroureterectomy results in a statistically significant increase in eGFR compared to post-NAC levels [MD = 15.56; 95% CI, 11.51-19.61] (Supplementary Figure 10). We observed statistically significant heterogeneity [*I* ^2^ = 71.45%, *P *= .01]; however, the leave-one-out sensitivity analysis revealed no significant change in the reported effect estimate (Supplementary Figure 11).

#### NAC vs. control:

**Supplementary Figure 12.**
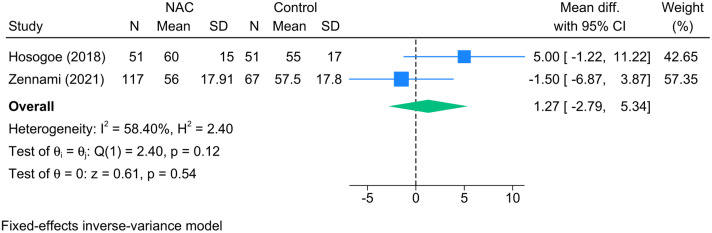
Forest plot showing the difference in eGFR levels between the neoadjuvant chemotherapy and control groups.

Two studies compared the eGFR levels between the NAC and the control groups. No statistically significant change was observed in eGFR between patients who received NAC and those who did not (control group) [MD = 1.27; 95% CI, −2.79-5.34] (Supplementary Figure 12). The observed statistical heterogeneity was insignificant [*I* ^2^ = 58.40%, *P *= .12].

### Safety profile of Neoadjuvant Chemotherapy

#### Overall complications:

**Supplementary Figure 13.**
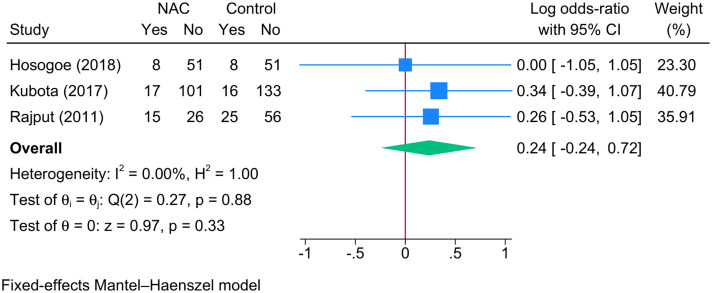
Forest plot showing the difference in overall complications between the neoadjuvant chemotherapy and control groups.

Complications were reported in 4 studies with a pooled rate of 25% [95% CI, 7-43]. Compared to the control group, NAC was not associated with a significant difference in the risk of overall complications [3 studies, logOR = 0.24; 95% CI, −0.24-0.72] (Supplementary Figure 13). No heterogeneity was observed [*I* ^2^ = 0%, *P *= .88].

#### Grade 3/4 complications:

**Supplementary Figure 14.**
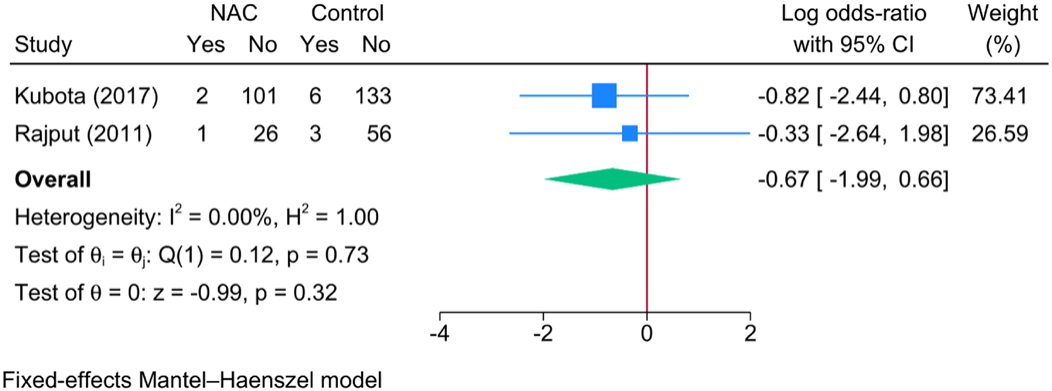
Forest plot showing the difference in grade 3-4 complications between the neoadjuvant chemotherapy and control groups.

Grade 3-4 complications were reported in 4 studies with a pooled rate of 7% [95% CI, 0-17%]. Compared to the control group, the NAC cohort was not associated with a significant difference in the risk of grade 3-4 complications [2 studies, logOR = −0.67; 95% CI, −1.99-0.66] (Supplementary Figure 14). No heterogeneity was observed [*I* ^2^ = 0%, *P *= .73].

## Discussion

In this comprehensive meta-analysis, we evaluated the impact of NAC on patients with UTUC undergoing RNU. Our findings elucidate the potential benefits and drawbacks of NAC in this patient population, offering valuable insights for clinical decision-making.

### Pathological Outcome

The observed 10% rate of CPR in NAC patients underscores the potential of NAC to eradicate the microscopic disease, a finding consistent with previous studies on bladder cancer.^32^ The significantly higher odds of CPR in NAC patients compared to the control group further affirms the therapeutic advantage of NAC. However, the substantial heterogeneity detected in our analysis indicates variability in response, necessitating individualized patient assessment and highlighting the need for biomarkers to predict the response to NAC. With a 48% rate of PPR in NAC patients, our findings indicate that nearly half of the patients experience a substantial reduction in tumor burden. This is a promising result, as PPR is often associated with improved surgical outcomes and may contribute to a better long-term prognosis. The lack of a control group comparison for PPR is a limitation in our study, and future research should address this gap to provide a more comprehensive understanding of NAC’s impact.

Our data showed that 42% of patients experienced pathological downstaging following NAC, and the odds of downstaging were significantly higher in the NAC group compared to the controls. This suggests that NAC effectively downstages the tumor, potentially making surgical resection more feasible, thereby improving patient outcomes. The observed heterogeneity underscores the variability in response and highlights the need for further research to elucidate the factors influencing downstaging. The observed 31% rate of advanced disease (pT3 or pT4) post-NAC, along with the significantly lower risk compared to the control group, suggests that NAC plays a crucial role in reducing the likelihood of advanced disease at the time of surgery. This is a key finding, as advanced disease is often associated with a poorer prognosis. The significant heterogeneity observed indicates variability in the study populations and highlights the need for standardized reporting and patient selection criteria.

Our study reports a 6% rate of PSM in NAC patients, with no significant difference observed compared to the control group. This low rate of PSM is encouraging, as it suggests that NAC does not increase the risk of incomplete resection. The lack of heterogeneity in these results adds to their reliability. The 18% rate of pN+ in our study is a critical finding, as lymph node involvement is a known prognostic factor in UTUC. The absence of a significant difference between NAC and control groups in terms of pN+ risk suggests that NAC may not have a substantial impact on lymph node metastasis. The lack of heterogeneity in these results suggests consistency across different studies. Our analysis revealed a 24% rate of LVI in NAC patients, with no significant difference in risk compared to the control group. LVI is a known adverse prognostic factor, and the lack of a significant impact of NAC on LVI risk highlights an area for future research, particularly in terms of optimizing NAC regimens and identifying patients most likely to benefit.

### Survival

Our findings demonstrate a significant reduction in the risk of mortality at 5 years in patients receiving NAC compared to the control group. This highlights the short-term benefits of NAC in improving patient survival. However, it is crucial to note that this survival advantage diminishes over time, with no significant difference observed in the long-term. This pattern of initial benefit followed by a convergence in mortality rates aligns with the previous studies on neoadjuvant therapies in urothelial carcinoma,^8,33^ emphasizing the need for ongoing patient monitoring and consideration of additional therapeutic interventions over time. The trend observed in disease recurrence mirrors that of mortality, with a notable reduction in risk at 5 years post-NAC, but no sustained long-term benefit. This suggests that while NAC may effectively reduce the immediate risk of disease recurrence, its impact does not persist over a longer period. This finding is consistent with existing literature,^7,34^ highlighting the potential for disease recurrence even after initial successful NAC treatment.

Our study indicates a clear benefit of NAC in terms of OS, particularly within the first 10 years of treatment. Patients receiving NAC exhibited significantly higher odds of OS at both 5- and 10-years post-treatment compared to the control group. This reinforces the role of NAC not only in reducing the tumor burden but also in enhancing patient survival. These results are in line with previous studies that have demonstrated the survival benefits of NAC in urothelial carcinoma patients.^34,35^ The stability in CSS rates across various follow-up periods in our study suggests that NAC has a sustained impact on cancer-specific mortality. Compared to the control group, patients receiving NAC had significantly higher odds of CSS at all observed time points, underlining the long-term benefits of NAC in specifically targeting urothelial carcinoma. This is a particularly noteworthy finding, as it is in contrast with the patterns observed in overall mortality and disease recurrence.

Our analysis of PFS revealed a relatively unexpected trend, with higher rates observed at 10 years compared to 5 years post-NAC. This could suggest a delayed benefit of NAC in preventing disease progression, a finding that warrants further investigation. Compared to the control group, NAC was associated with significantly higher odds of PFS at both 5 and 10 years, supporting its role in maintaining disease stability over time. The RFS rates observed in our study, along with the lack of a significant difference between NAC and control at 5 years, suggest a nuanced impact of NAC on recurrence. The limited data available for longer-term RFS highlights a gap in the current literature and underscores the need for extended follow-up in future studies to fully elucidate the long-term impact of NAC on recurrence.

### Renal Function

The impact of NAC and RNU on renal function, as assessed by eGFR, is a critical aspect of UTUC management. Our study revealed no significant change in eGFR levels following NAC administration. This finding is reassuring, as it suggests that NAC does not adversely affect renal function in the short term. This is consistent with previous literature,^36^ where the impact of chemotherapy on renal function has been a subject of ongoing investigation, with varying results depending on the chemotherapy agents used and the patient population studied.

The significant increase in eGFR observed from pre-NAC to post-RNU is an intriguing finding. Typically, surgeons might expect a decrease in renal function following surgical intervention, particularly a procedure as extensive as RNU. However, our results suggest a potential preservation or even improvement in renal function. While the observed heterogeneity indicates variability across studies, the leave-one-out sensitivity analysis confirms the robustness of this result. This finding may reflect the complex interplay between tumor burden, renal function, and the effects of surgical intervention, pointing to the need for a nuanced interpretation and further research to elucidate these dynamics. A similar observation was noted between pre-NAC and post-RNU eGFR levels. Additionally, the use of NAC was not associated with a significant difference in eGFR from the control. This finding suggests that NAC, in itself, does not confer an advantage or a disadvantage in terms of renal function when compared to the control group. However, this finding is limited by the factor of time, and due to the lack of data on eGFR assessment timepoint, analysis was not feasible.

### Safety Profile

The safety and tolerability of NAC and subsequent RNU are paramount considerations in the management of UTUC. Our study provides a comprehensive analysis of the complications associated with these interventions, contributing valuable data to the existing body of knowledge. Our findings indicate a pooled rate of 25% for overall complications associated with NAC and RNU. This rate is within the expected range, considering the complexities and potential risks of chemotherapy and major surgical procedures. Importantly, our analysis revealed no significant difference in the risk of overall complications between patients receiving NAC and the control group. This is a critical finding, as it suggests that the addition of NAC to the treatment regimen does not increase the overall risk of complications. Our findings are in line with previous research,^37^ which has found NAC to be a safe and well-tolerated intervention in UTUC. When focusing on more severe complications (grade 3/4), our study reported a pooled rate of 7%. This relatively low rate of serious complications is reassuring and supports the safety profile of NAC in conjunction with RNU. Moreover, we found no significant difference in the risk of Grade 3/4 complications between the NAC group and the control group, further affirming the safety of NAC in this clinical context. The absence of heterogeneity in these results adds to their robustness.

Our meta-analysis highlights the potential benefits of NAC in enhancing pathological outcomes and possibly improving survival in UTUC patients undergoing RNU. The variability in response and associated complications underscore the importance of careful patient selection and tailored treatment approaches. Our findings pave the way for future research to optimize NAC strategies, ultimately improving patient outcomes in UTUC.

## Limitations and Future Directions

While our study provides valuable insights, it is not without limitations. The heterogeneity observed in some analyses may stem from variations in study design, patient populations, or NAC regimens. Additionally, the retrospective nature of many included studies may introduce bias. Future prospective studies with standardized protocols are essential to validate our findings and refine NAC utilization in UTUC. Moreover, future studies should focus on studying regimen-focused, dose-dependent responses.

## Figures and Tables

**Table 1.** Characteristics of studies discussing neoadjuvant chemotherapy in UTUC patients

Author (YOP)	Design	Country	Sample		Age				Male		AC		Tumor Location							
			NAC	Control	NAC		Control		NAC	Control	NAC	Control	Kidney		Renal Pelvis		Ureter		Multifocal	
					mean	SD	mean	SD					NAC	Control	NAC	Control	NAC	Control	NAC	Control
Adibi (2022) ^11^3	RC	USA	126	68	68#	32-88#	-	-	81	-	-	-	-	-	57	-	50	-	11	-
Almassi (2018) ^12^3	RC	USA	260	5914	67.5	9.69	73	10.38	156	3491	-	-	168		3320		92	2594	-	-
Chen (2020) ^13^3	RC	China	37	37	60.49	5.83	61.95	5.41	22	18	0	0	19	23	-	-	18	14	-	-
Coleman (2023) ^14^3	Phase II trial	USA	57	-	61.75	11.3	-	-	-	-	-	-	-	-	-	-	-	-	-	-
Gabriel (2023) ^15^3	RC	France	24	-	57.13	9.17	-	-	-	-	-	-	-	-	-	-	-	-	-	-
Ghandour (2021) ^16^3	RC	USA	106	-	67.11	9.24	-	-	76	-	-	-	-	-	47	-	25	-	26	-
Grossmann (2022) ^17^3	RC	Austria	164	-	68	63.73	-	-	113	-	10	164	-	-	-	-	-	-	-	-
Hosogoe (2018) ^18^3	RC	Japan	51	51	70	9.6	70	9.1	40	38	-	-	0	0	20	17	31	34	0	0
Kobayashi (2016) ^19^3	RC	Japan	24	31	67	15.3	72	11.5	15	21	-	-	-	-	18	24	6	4	0	3
Kubota (2017) ^20^3	RC	Japan	101	133	70	9.5	71	8.9	70	85	0	0	-	-	31	61	64	61	6	11
Labbate (2022) ^21^3	RC	USA	152	-	69	12.6	-	-	103	-	-	-	-	-	73	-	59	-	21	-
Liao (2018) ^22^3	RC	USA	32	208	-	-	-	-	-	-	-	-	-	-	-	-	-	-	-	-
Margulis (2020) ^23^3	Phase II trial	USA	35	-	65.13	10.46	-	-	27	-	-	-	-	-	-	-	-	-	-	-
Miyake (2019) ^24^3	RC	Japan	32	-	69	8.9	-	-	22	-	32	-	-	-	-	-	32	-	-	-
Porten (2014) ^25^3	RC	USA	31	81	67.32	12.9	67.35	9.08	19	48	0	20	-	-	21	47	9	24	-	-
Rajput (2011) ^27^3	RC	USA	26	56	70.97	8.08	74.18	9.18	17	34	-	-	-	-	14	24	20	23	2	9
Singla (2020) ^28^3	RC	USA	43	191	68.65	8.44	68.65	11.2	29	125	0	0	28	106	-	-	8	60	10	68
Venkat (2021) ^29^3	RC	USA	309	5297	73*	(65.0 – 80.0)*	67*	(61.0 – 73.0)*	3141	196	-	-	-	-	-	-	122	232	-	-
Yu (2023) ^30^3	RC	Taiwan	84	-	-	-	-	-	38	-	17	-	-	-	35	-	29	3	37	-
Zennami (2021) ^31^3	RC	Japan	117	67	-	-	-	-	-	-	-	-	-	-	-	-	-	-	-	-
Pradere (2021) ^26^3	RC	Austria	172	-	68	7.48	-	-	122	-	-	-	-	-	-	-	-	-	33	-

*Data were reported as median and interquartile range; -indicates that the data were not reported; #data were reported as median and range.

AC, adjuvant chemotherapy; NAC, neoadjuvant chemotherapy; RC, retrospective cohort; UTUC, upper tract urothelial cancer; YOP, year of publication.

**Table 2.** Regimen, Dose, and the Number of Cycles of Administered Neoadjuvant Chemotherapy in Upper Tract Urothelial Cancer Patients

Author (YOP)	Regimen	Number	Dose	Cycles
Adibi (2022) ^11^3	CGI	13	-	4
	GCis	15	-	4
	ddMVAC	62	-	4
	Multi	17	-	4
	GTA	19	-	4
Almassi (2018) ^12^3	-	-	-	-
Chen (2020) ^13^3	Gemcitabine 1250 mg/m^2^3	-	2 (on days 1 and 8)	2-4
	Cisplatin 75 mg/m^2^3	-	3 (on days 1–3)	2-4
Coleman (2023) ^14^3	-	-	-	-
Gabriel (2023) ^15^3	-	-	-	-
Ghandour (2021) ^16^3	GCis	-	-	-
	MVAC	-	-	-
Grossmann (2022) ^17^3	GC	66	-	(1-4)
	MVAC	66	-	(1-4)
	Non-cisplatin	32	-	(1-4)
Hosogoe (2018) ^18^3	GCis	16	Days 1, 8, and 15	2-4
	GCb	35	Days 1, 8, and 15	2-4
Kobayashi (2016) ^19^3	-	-	-	-
Kubota (2017) ^20^3	GCis	21	Day 2 every 3 weeks	2-4
	GCb	76	Day 2 every 3 weeks	2-4
Labbate (2022) ^21^3	-	-	-	-
Liao (2018) ^22^3	Methotrexate + vinblastine + doxorubicin + cisplatin	12	-	4 ± 1
	Gemcitabine + cisplatin	17	-	-
	Gemcitabine + cisplatin + bevacizumab	1	-	-
	Gemcitabine + docetaxel	1	-	-
	Gemcitabine	1	-	-
Margulis (2020) ^23^3	Accelerated methotrexate + vinblastine + doxorubicin + cisplatin	29	-	4
Miyake (2019) ^24^3	GCis	24	-	2-3
	MVAC	3	-	2-3
	GCb	6	-	4
	Platinum-based regimens including carboplatin	5	-	4
Porten (2014) ^25^3	MVAC	21	-	4.35 ± 0.8
Rajput (2011) ^27^3	MVAC	6	-	4 ± 1.5
	GCis + ifosfamide	6	-	-
	MVAC + Avastin	5	-	-
	GCis	2	-	-
	MVAC	2	-	-
Singla (2020) ^28^3	Cisplatin-based chemotherapy	43	-	-
Venkat (2021) ^29^3	-	-	-	-
Yu (2023) ^30^3	Cisplatin-based	44	-	-
	Carboplatin-based	22	-	-
	Others^*^3	18	-	-
Zennami (2021) ^31^3	GCis or MVAC	107	-	1-2
	GCb	10	-	1-2
Pradere (2021) ^26^3	Cisplatin-based	162	-	-
	Carboplatin-based	10	-	-

GCb, gemcitabine + carboplatin; GCis, gemcitabine + cisplatin; MVAC, methotrexate + vinblastine + adriamycin + cisplatin; CGI: cisplatin + gemcitabine + ifosfamide; ddMVAC: dose-dense methotrexate + vinblastine + adriamycin + cisplatin; GTA: gmcitabine + paclitaxel + doxorubicin; UTUC, upper tract urothelial carcinoma; YOP, year of publication.

*The details were not provided in the original study.

**Table 3.** The Methodological Quality of Included Studies as Per the Newcastle-Ottawa Scale for Observational Studies

Author (YOP)	Selection	Comparability	Outcome	Overall Quality
Adibi (2022) ^11^3	3	2	3	Good
Almassi (2018) ^12^3	3	2	3	Good
Chen (2020) ^13^3	3	2	3	Good
Coleman (2023) ^14^3	3	1	3	Fair
Gabriel (2023) ^15^3	3	1	3	Fair
Ghandour (2021) ^16^3	3	1	3	Fair
Grossmann (2022) ^17^3	3	1	3	Fair
Hosogoe (2018) ^18^3	3	2	3	Good
Kobayashi (2016) ^19^3	3	2	3	Good
Kubota (2017) ^20^3	3	2	3	Good
Labbate (2022) ^21^3	3	1	3	Fair
Liao (2018) ^22^3	3	2	3	Good
Margulis (2020) ^23^3	3	1	3	Fair
Miyake (2019) ^24^3	3	1	3	Fair
Porten (2014) ^25^3	3	2	3	Good
Rajput (2011) ^27^3	3	2	3	Good
Singla (2020) ^28^3	3	2	3	Good
Venkat (2021) ^29^3	3	2	3	Good
Yu (2023) ^30^3	3	1	3	Fair
Zennami (2021) ^31^3	3	2	3	Good
Pradere (2021) ^26^3	3	1	3	Fair

YOP, year of publication.

**Supplementary Table 1.** The detailed search query employed in the database search

Database	Number	Search Query		Results
PubMed [Date of search: Sept 11, 2022]				
3	#1	“upper tract” OR “upper urinary tract”		11001
	#2	“urothelial cancer” OR “urothelial carcinoma” OR “urothelial neoplasm” OR “transitional cell carcinoma”		32187
	#3	#1 AND #2		4062
	#4	UTUC		1544
	#5	#3 OR #4		4136
	#6	Nephroureterectomy OR “Nephroureterectomy”[Mesh] OR “Ureteral Neoplasms”[Mesh]		7619
	#7	Chemotherapy		47409
	#8	neoadjuvant OR neo-adjuvant OR “Neoadjuvant Therapy”[Mesh] OR preoperative		843749
	#9	#5 AND #6 AND #7 AND #8		432
Scopus [Date of search: Sept 11, 2022]				
3	#1	ALL (“upper tract”) OR ALL (“upper urinary tract”)		37938
	#2	ALL (“urothelial cancer”) OR ALL (“urothelial carcinoma”) OR ALL (“urothelial neoplasm”) OR ALL (“transitional cell carcinoma”)		113292
	#3	#1 AND #2		14597
	#4	ALL (UTUC)		1874
	#5	#3 OR #4		14632
	#6	ALL (Nephroureterectomy)		9546
	#7	ALL (chemotherapy)		2527998
	#8	ALL (neoadjuvant) OR ALL (preoperative) OR ALL (neo-adjuvant)		1725068
	#9	#5 AND #6 AND #7 AND #8		1948
Web of Science [Date of search: Sept 11, 2022]				
3	#1	ALL=”upper tract” OR ALL=”upper urinary tract”		11338
	#2	ALL=”urothelial cancer” OR ALL=”urothelial carcinoma” OR ALL=”urothelial neoplasm” OR ALL=”transitional cell carcinoma”		34310
	#3	#1 AND #2		5325
	#4	ALL=UTUC		1564
	#5	#3 OR #4		5415
	#6	ALL=Nephroureterectomy		4153
	#7	ALL=chemotherapy		669424
	#8	ALL=neoadjuvant OR ALL=preoperative OR ALL=neo-adjuvant		547972
	#9	#5 AND #6 AND #7 AND #8		472
CENTRAL [Date of search: Sept 11, 2022]				
3	#1	“upper tract” OR “upper urinary tract”		626
	#2	“urothelial cancer” OR “urothelial carcinoma” OR “urothelial neoplasm” OR “transitional cell carcinoma”		1758
	#3	#1 AND #2		207
	#4	UTUC		84
	#5	#3 OR #4		208
	#6	Nephroureterectomy		139
	#7	chemotherapy		96939
	#8	neoadjuvant OR preoperative OR neo-adjuvant		88431
	#9	#5 AND #6 AND #7 AND #8 [Trials only]		46
Google Scholar [Date of search: Sept 11, 2022]				
3	With all of the words	chemotherapy nephroureterectomy UTUC		
	With the exact phrase	-	3	
	With at least one of the words	neoadjuvant preoperative pre-operative neo-adjuvant		
	Total	Only the first 200 were retrieved and screened		200
